# Management of May Thurner Syndrome in Pregnant Patients

**DOI:** 10.3390/jcdd9120410

**Published:** 2022-11-23

**Authors:** Tabitha L. Schrufer-Poland, Karen Florio, Anna Grodzinsky, John J. Borsa, Laura Schmidt

**Affiliations:** 1 Maternal and Fetal Medicine, Saint Luke’s Health System, Kansas City, MO 64111, USA; 2 Cardiovascular Outcomes Research Department, Saint Luke’s Health System, Kansas City, MO 64111, USA; 3 Department of Radiology, Saint Luke’s Health System, Kansas City, MO 64111, USA; 4 Department of Radiology, University of Missouri Kansas City, Kansas City, MO 64108, USA

**Keywords:** May-Thurner, May Thurner, pregnancy, Cockett Syndrome, iliac compression, venous thromboembolism, anticoagulation

## Abstract

May Thurner Syndrome contributes to thromboembolic disease and can cause significant morbidity in pregnant patients secondary to exaggerated anatomic relationships and physiologic changes in the hematologic system favoring thrombogenesis. Because this condition is both underrecognized and underreported, management in pregnant and postpartum patients is based on expert opinion without any formal evidence-based guidance. Herein, we review five pregnancies in four patients with May Thurner Syndrome and general management strategies. Through collaborative and multidisciplinary care, patients with May Thurner Syndrome can be safely and successfully managed during pregnancy and the postpartum period with appropriate anticoagulation.

## 1. Introduction

**Figure 1:**
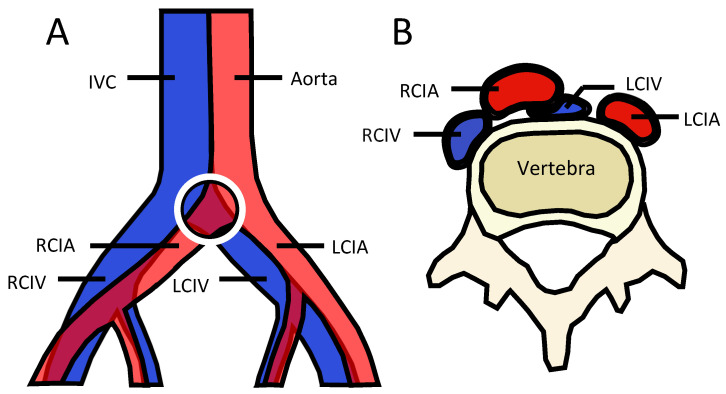
Illustration of May Thurner Anatomy. (**A**) Coronal and (**B**) Transverse illustrations demonstrating the most common conformation of May Thurner anatomy with compression of the LCIV by the anterior crossing RCIA.

May Thurner Syndrome (MTS) is an anatomic condition resulting from extrinsic venous compression of the left common iliac vein (LCIV) between the right common iliac artery (RCIA) and the lumbar vertebra (Figure 1). Over time, pulsatile compression results in vascular remodeling, leading to thrombosis, chronic venous insufficiency, and associated sequelae [1].

May Thurner anatomy has been implicated in 2–5% as the underlying etiology of patients being evaluated for symptomatic lower extremity venous disorder, and up to 20% of patients presenting with thromboembolism, particularly those presenting with extensive left-sided, or recurrent disease [2,3]. During pregnancy, physiologic compression of the inferior vena cava (IVC) and LCIV by both the gravid uterus, and the exaggerated lordosis associated with pregnancy, can exacerbate the compression associated with MTS, resulting in a higher risk of thrombosis in this patient population.

Although management is well-defined in the non-pregnant population, there is a paucity of literature regarding diagnosis, management, and outcomes of patients with MTS during pregnancy. Herein we present a case series describing management and outcomes in 4 patients with MTS, over 5 pregnancies, as well as a review of management, and special considerations for care of this unique patient population.

## 2. Cases

**Figure 2:**
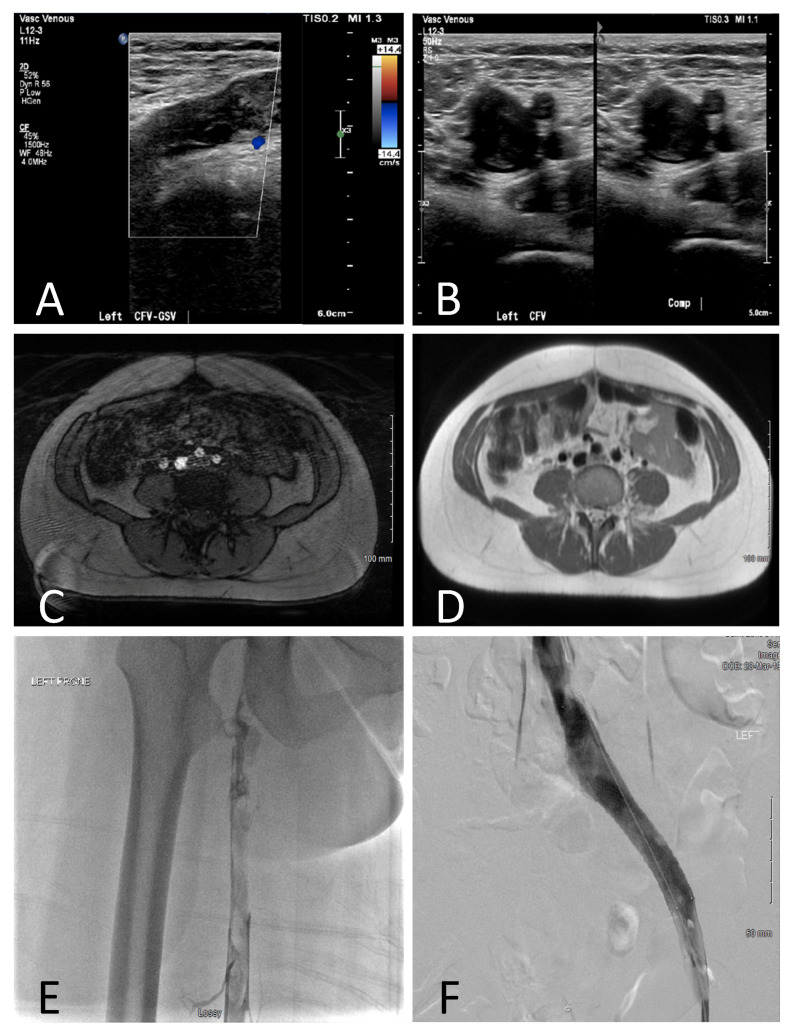
Diagnostic Imaging from Patient 1. Duplex/Doppler ultrasound demonstrating occlusive thrombus in the CFV-GSV (**A**). Coronal T1-weighted (**B**) and axial T2-weighed MRA/V (**C**) demonstrating compression of the LCIV between the spine and RCIA, with extensive thrombus (arrows). IVUS demonstrating occlusive thrombus (**D**). Venography before (**E**) and after (**F**) stent placement postpartum.

Patient 1 is a 29-year-old gravida 2 who presented at 9 weeks’ gestation with left lower extremity pain and swelling. Doppler ultrasound demonstrated extensive thrombus extending from the left posterior tibial and peroneal veins into the left common femoral vein (CFV), involving the left greater saphenous vein (GSV) (Figure 2A). MRI/MRA/MRV of the pelvis demonstrated extension throughout the external and common iliac veins, and extrinsic compression of the left common iliac vein (LVIC), as well as extension into the infrarenal IVC (Figure 2B,C). She was started on intravenous unfractionated heparin (UFH). A venogram with intravenous ultrasound (IVUS) was performed with placement of an infusion catheter across the thrombosed segments, as can be visualized in Figure 2D,E. Catheter-directed thrombolysis (CDT) was performed with tissue plasminogen activator (tPA) infusion (0.5–1 mg/h) through the catheter with UFH (400 units/h) through the access sheath for 48 h. Daily venograms with IVUS were performed to assess possible resolution, with balloon maceration at each instance. After 48 h there was complete patency of the area with restoration of flow. Total recorded radiation dose was 16 mGy, with 4.6 min of fluoroscopy, with shielding. Adjusted dose low molecular weight heparin (LMWH) was continued for the duration of the pregnancy and held 24 h prior to her scheduled delivery. She delivered vaginally at term without complications. LMWH was resumed 12 h after delivery for an additional 6 weeks. Venogram was performed 8 weeks postpartum, with placement of a 14 × 90 mm self-expanding stent in the LCIV as per the decision of the interventional team following multidisciplinary discussion (Figure 2F).

Patient 2 is a 28-year-old who was diagnosed with MTS prior to pregnancy, after experiencing an extensive left lower extremity deep venous thrombosis (DVT), 4 weeks after initiation of an etonogestrel/ethinyl estradiol vaginal ring. Following diagnosis, she was placed on rivaroxaban for 4 months. She had a follow-up ultrasound 10 months after initiation of therapy, which was significant for moderate asymmetric narrowing of the LCIV, with increased right-sided velocities, suggestive of MTS. CT pelvis demonstrated compression of the LCIV. Placement of an LCIV stent was recommended per the managing team; however, the patient became pregnant prior to follow-up. During pregnancy, she was managed with prophylactic LMWH, at a dose of 40 mg daily, and transitioned to UFH at 36 weeks, per the primary managing team with patient-centered decision making. She delivered vaginally at term without complications. She had no obstetric or vascular complications during her pregnancy, but developed mild asymptomatic varicosities postpartum. She continued prophylactic LMWH until venography with stent placement 8 weeks postpartum, again per the recommendations of the managing team. Two 22 × 75 mm self-expanding stents were placed in the LCIV, followed by balloon dilation, via LCFV sheath. She continued LMWH for 30 additional days, with a plan for indefinite aspirin monotherapy. Three years after her stent placement, she became pregnant again. Repeat ultrasound demonstrated patency of both the stent and vein. She was managed with aspirin and prophylactic dose LMWH throughout the pregnancy and delivered vaginally at term, again without complications. She did not experience any obstetric nor venous complications, nor worsening of her venous insufficiency. LMWH was continued for 6 weeks postpartum, followed by indefinite aspirin monotherapy.

Patient 3 is a nulliparous 29-year-old who presented with left lower extremity discomfort and swelling. Initial evaluation was unremarkable and Doppler ultrasound was negative. She presented again 9 days later with phlegmasia cerula dolens of the left leg. Repeat imaging demonstrated extensive DVT from the posterior tibial and peroneal veins, propagated to the left external iliac vein (LEIV), involving all branches of the CFV. She has been on combined oral contraceptives since the age of 16, without any prior VTE events. She was started on intravenous UFH. Venogram with IVUS was performed via left popliteal vein (LPV) access for pharmacomechanical thrombectomy (PMT) and CDT with tPA (1 mg/h) infusion overnight. Subsequently, a 14 × 80 mm self-expanding stent was deployed into the LCIV, followed by balloon dilation and she was placed on rivaroxaban for 1 year. Two years later she complained of increasing left groin and lower back pain. A diagnostic ultrasound revealed venous scarring with non-compressibility of the LCIV, LCFV and GSV. CT pelvis exhibited occlusion of the stent with large pelvic collateral circulation, which was confirmed on venography. Multiple attempts for recanalization were unsuccessful and she resumed anticoagulation. Three years following her initial diagnosis she became pregnant with her first child. She was maintained on intermediate dose LMWH (40 mg twice daily) during pregnancy and was transitioned to UFH near term. She had a vaginal delivery at term without complications. There was no worsening of her venous insufficiency. Postpartum she was bridged to warfarin, with a plan to restart apixaban after completion of breastfeeding.

Patient 4, a 32-year-old gravida 3 presented with chronic pelvic pain and varicosities of the vulva and perineum that had developed during her second pregnancy. Bilateral Duplex venous mapping demonstrated extensive pelvic collateral circulation and widespread insufficiency consistent with MTS, which was confirmed with venography. She underwent stenting of both LCIV and the LEIV with two 20 × 90 mm self-expanding stents with balloon dilation and was discharged on rivaroxaban and aspirin with plan for outpatient follow-up in one month. However, twelve days after her stent placement she had a positive pregnancy test and was estimated to be 5 weeks’ gestation by her last menstrual dating. She was switched to prophylactic LMWH and continued on aspirin, with transition to UFH near term. She had a vaginal delivery at term without complications. She was restarted on prophylactic LMWH 12 h postpartum. However, on postpartum day 6 she developed a thrombus in the right gonadal vein, which was imaged and noted to be dangling into the IVC; the stents were noted to be grossly patent. Upon questioning, she reported missing only a single dose of LMWH. She was restarted on IV UFH and bridged to warfarin inpatient. CT venogram 2 months postpartum noted a thrombus with slit-like narrowing of the mid-femoral vein lumen distal to the stent, but following a multidisciplinary discussion was determined not to require intervention or treatment. She was continued on warfarin and aspirin with planned close follow-up. See Table 1 for summary of cases.

## 3. Discussion

MTS is an anatomic phenomenon most commonly arising as a result of extrinsic compression of the LCIV between the lumbar vertebrae and RCIA, which results in increased risk of thrombosis and venous insufficiency (Figure 1). Based on their observations, May and Thurner, and later, Cockett, proposed that chronic irritation of the endothelium of the vein compressed between the lumbar vertebra posteriorly, and the pulsatile artery anteriorly, results in vascular spur formation [1,4,5]. Over time, vascular remodeling leads to stasis, thrombogenesis, and venous hypertension with associated sequelae [4].

The exact prevalence of MTS is unknown; however, the syndrome has been reported in approximately 2–5% of patients who present with a symptomatic lower extremity venous disorder, and up to 20% of those presenting with acute DVT [2,3,6]. However, these are likely significant underestimates given the 20–30% prevalence of May Thurner anatomy in cadaveric subjects [1,7,8], and the 25–66% prevalence of LCIV narrowing noted in asymptomatic patients undergoing pelvic imaging for unrelated indications [9,10]. In pregnant patients the incidence of May Thurner anatomy-related thrombosis is likely even higher given the exaggerated lordosis and compression from the gravid uterus. Therefore, some authorities, including the American College of Obstetricians and Gynecologists (ACOG), have advocated a need to rule out MTS in pregnant women with acute left lower extremity DVT [11]. Regardless, there is a lack of evidence-based guidance on management during pregnancy.

### 3.1. Diagnosis

Although several attempts have been made to develop and standardize anatomic criteria for MTS, there is still a lack of diagnostic guidance [12,13,14]. Diagnostic parameters dependent upon 2-dimensional imaging, operator dependence, and patient characteristics, are fraught with confounding, and as such pose significant barriers to the development of diagnostic criteria. Additionally, there have been no well-designed studies to address the degree of stenosis or intravascular pathology that is clinically relevant, which may inadvertently result in overdiagnosis, with risk of exposure to potentially unnecessary invasive procedure and intervention [15]. In fact, many primary and secondary signs of compression can be demonstrated in healthy volunteers. In one study, 80% of study participants had at least 2 radiographic signs of compression by venography [16]. As such, diagnosis of MTS as the causative etiology for venous pathology requires high clinical suspicion, as well as a combination of clinical assessment and multimodal diagnostic imaging techniques. At this time, diagnosis is suspected by noninvasive imaging techniques including ultrasound and MRI and confirmed on invasive imaging or at the time of intervention, based on the surgeon’s assessment of extrinsic compression. In pregnant patients, anatomic relationships, unique imaging constraints, and concern regarding radiation exposure make definitive diagnosis of MTS as a potential underlying etiology even more complex, if not impossible. Therefore, follow-up after the conclusion of pregnancy is necessitated for those with suspected MTS based on imaging performed during pregnancy.

#### 3.1.1. Ultrasound

While ultrasound should be considered a first line tool for diagnosis of DVT in pregnancy, discrimination of MTS as the causative etiology cannot be definitively made by ultrasound. Secondary signs of MTS include stenosis of the iliofemoral vessels, turbulent flow, extensive collateral vessels, and evidence of venous insufficiency. However, a definitive point of compression may not be visualized. Nonetheless, this imaging tool provides useful, noninvasive information that allows for risk stratification. In patients with primary evidence for compression, postural change with reassessment may help to distinguish illusory with flow recovery from true (persistent) compression [17]. In the obstetric population, DVT is significantly more likely to occur proximally, with a high predilection for involvement of the iliofemoral vessels [11,18,19,20]. Although iliac Duplex imaging can provide a dynamic and functional assessment of the iliofemoral veins [3], above the inguinal plane, ultrasound has diminished sensitivity, and can be significantly limited by body habitus, depth, general lack of compressibility-particularly of retroperitoneal structures. In pregnant and postpartum patients, the presence of a gravid or enlarged uterus can result in a high rate of non-visualization. Several studies have documented that the common iliac vein may be non- or inadequately visualized in almost 50% of patients, and the external iliac vein non- or inadequately visualized in up to 20% of patients, even with experienced sonographers [21,22,23]. Pregnant patients, in whom a more conservative approach to diagnosis is warranted secondary to the risk for severe morbidity, benefit from imaging that allows for evaluation with higher specificity and discrimination of vascular anatomic relationships.

#### 3.1.2. Magnetic Resonance Imaging

Magnetic resonance imaging (MRI) with MR venography (MRV) is an attractive adjuvant modality for pregnant patients with suspected DVT with negative ultrasound, as well as an excellent modality to help delineate anatomic relationships necessary for diagnosis of MTS in nonpregnant patients. MR is considered safe, without conclusive evidence that there are deleterious effects on the fetus/child [24,25]. Theoretical concerns of auditory damage with first trimester exposure have not been demonstrated in animal or small human studies [26,27]. In a study of the extent of thrombosis in 27 pregnant patients, MR consistently outperformed ultrasound, detecting a higher number of DVTs and in 2/3 of cases, more proximal extent of disease. Information provided by MR is significantly enhanced with the use of contrast, which is not recommended in pregnant patients [28]. In these patients time of flight with additional sequencing and magnetic resonance direct thrombus imaging (MRDTI) can provide additional information without the need for contrast [29].

#### 3.1.3. Computed Tomography and Venography

CT is a useful diagnostic tool for evaluation of patients with MTS, for assessment of anatomic relationships, presurgical planning, and for diagnosis of acute and subacute thromboses. Studies in nonpregnant patients demonstrate a sensitivity and specificity for the detection of DVT which is similar, if not superior, to other methods [30,31]. Compared to conventional venography, CT venography (CTV) is widely available, less invasive and lacks operator dependence. Additionally, CT can evaluate vertebral pathology, and allows for measurement of the degree of maximal lordosis, accurately describing anatomic relationships between the vessels and spine. CT can also be used to assess collateral circulation and vessel tortuosity. Delineation of these relationships can facilitate preoperative planning for stent placement [32]. Conventional contrast venography has historically been regarded as the definitive diagnostic imaging modality for detection of venous pathology, and has been the standard to which less invasive testing has been compared [33]. This technique allows for real-time evaluation of pressure gradients across stenotic areas and assessment of collateral circulation [34,35]. However, like other methods, there are no clearly defined criteria to identify clinically significant lesions. During pregnancy, utilization of conventional venography has been limited due to its invasive nature and the radiation associated with the technique, in lieu of alternative imaging modalities, or deferment to the postpartum period for definitive imaging and therapy. CTV, however, is associated with both radiation exposure and, depending on technique, high dose contrast exposure, to allow for appropriate opacification of the central veins. Therefore, MR should be the ancillary diagnostic test of choice in pregnant patients.

#### 3.1.4. Intravenous Ultrasound

IVUS has recently been advocated as a gold standard for diagnosis for MTS, replacing conventional venography alone in this role, both in superior sensitivity and safety [33,36]. This modality has become an integral part of evaluation of stenosis at the time of intervention, as well as planning for stent deployment (to evaluate vessel caliber and pathology, guide appropriate size and type of stent, and specific location for placement). Additionally, this technique enables assessment of vessels that are difficult to assess due to anatomic position, severe occlusion or the presence extensive collateral circulation, and can assess intravascular pathology such as webs, trabeculations, spurs, and vessel wall thickening. Therefore, IVUS is associated with higher sensitivity than conventional venography. IVUS also serves as a useful adjuvant in evaluation of later development of stent compression and restenosis [3]. Finally, use of IVUS significantly limits the amount of radiation associated with concurrent fluoroscopic guidance.

### 3.2. Radiation Exposure

Although not the focus of this review, concern regarding radiation exposure is a major barrier to diagnostic and treatment options offered to pregnant patients. When feasible, ultrasound and non-contrast MR should be utilized when imaging is necessitated; however, radiation exposure associated with radiography, nuclear medicine, or CT is generally well below the threshold anticipated to cause fetal harm. Therefore, when these modalities are necessitated for logistic or diagnostic reasons, they should not be withheld from pregnant patients [27]. Endovascular techniques and thrombolytic therapies have also been safely performed in pregnant patients; we and others have reported radiation doses that are well below the threshold anticipated to cause fetal harm [37,38]. With appropriate positioning, shielding, and procedural considerations, such as utilization of intravenous ultrasound, these procedures can be safely performed in pregnant patients at any gestational age, and therefore should not be withheld from pregnant patients when clinically indicated.

### 3.3. Surgical Management

#### 3.3.1. Endovascular Therapy

Minimally invasive techniques, now in combination with localized fibrinolytic therapy, have become the standard of care of management of MTS in the nonpregnant population, even in the case of limb-threatening disease. Current Society for Vascular Surgery guidelines recommend the use of endovascular therapy for primary management, as compared with anticoagulation alone, catheter-directed therapy is associated with significant reduction in long-term complications associated with PTS in the non-pregnant population (up to 90% develop PTS with anticoagulation alone, compared, to less than 10% with endovascular therapy) [30]. Percutaneous aspiration thrombectomy (PAT) utilizes ultrasound or fluoroscopic guidance to deliver manual negative pressure via syringe or suction to aspirate the thrombus. This method may be used in combination with more aggressive therapies (PMT or pharmacomechanical thrombectomy), including CDT, in which a thrombolytic agent is delivered directly to the area of thrombus. This technique increases local dose and duration of medication exposure, and thus efficacy and safety of thrombolysis. Often balloon dilation of the area is concurrently employed to allow for adequate space for delivery of therapy and correction of narrowing. An overview of endovascular techniques has previously been described [3].

Endovascular procedures have been safely utilized in pregnant and postpartum patients [37,38,39,40,41,42,43]; however, studies of long-term follow-up are significantly limited. In a study of 11 pregnant patients who underwent endovascular therapy for proximal DVT, technical success was achieved in all patients. Seventy-three percent (8/11) of patients had stents placed at the time of the procedure for residual stenosis and all continued their pregnancies, which were uneventful. Follow-up for a mean of 15.6 months revealed normal valve function and patent veins in 91% (10/11) [38]. In another study of 11 patients, 3 of whom had a diagnosis of MTS, technical success was achieved in >80% of patients; 73% required stent placement for residual stenosis. Three patients continued their pregnancies, which were uneventful. Patients were followed for median 20 months, although a second procedure was required in 2 patients for re-thrombosis, there were no reports of PTS [37]. Patient 1, presented herein, was diagnosed with MTS with extensive clot burden and phlegmasia cerulea dolens in the first trimester. After multidisciplinary conference and patient counseling, the patient was managed with a combination of CDT/PMT without adverse sequelae. The total recorded radiation dose during her procedure was well-within acceptable radiation dosage for pregnancy. Given the successes in these small case studies and our own experience, we believe management with CDT/PMT is reasonable for pregnant patients with MTS with extensive clot burden, and should be considered for those presenting with limb-threatening ischemia. As such, open thrombectomy should be considered only when more conservative approaches fail, in the setting of life- or limb-threatening ischemia [30]. Historically open procedures have been associated with high rates of maternal and fetal morbidity and mortality, as well as high re-operation rates [44].

#### 3.3.2. Vascular Stent Placement

Compared to balloon angioplasty or thrombectomy alone, and especially compared to anticoagulation alone, long-term venous patency rates associated with stenting are higher, with a lower risk of re-thrombosis, more complete resolution of thrombus, and lower risk for PTS, with reduced PTS severity [45,46]. As such, current Society for Vascular Surgery guidelines recommend the use of self-expanding stents for treatment of underlying compression or obstructive lesions following endovascular therapy in the nonpregnant population [30,47,48,49]. These recommendations are summarized nicely in a recent review by Mangla and Hamad [50]. Although invasive management carries inherent risk (occlusion, restenosis, thrombosis, and migration), the incidence of these complications is significantly lower than that of PTS [51,52]. Stent occlusion is a relatively uncommon phenomenon but may occur with greater frequency in those with associated thrombosis. In one large retrospective study, compression leading to stent obstruction was present in 3% of patients after stent placement; however, some degree of stent compression was noted in one-third of patients [47]. Restenosis occurs more commonly, with 5–20% of patients requiring reintervention for intrinsic occlusion resulting from intimal hyperplasia, calcification, and/or accelerated atherosclerosis [53,54]. Thrombosis occurs in 1–5%, and is associated with both occlusion and restenosis, as well as location of the stent [53,54]. Extension of the stent into the IVC can affect contralateral iliac flow resulting in increased risk of thrombosis on the contralateral side, as well as may influence migration and proximal stent deformation. Stent displacement/migration has been noted in 1–6.25% and may adversely affect function and can result in need for retrieval. Although exceedingly rare (4 reported cases), intracardiac migration has occurred, which necessitated open heart surgery in one case [52,55]. A significant number of stent failures and complications are related to technical issues with placement, including: lack of complete radial expansion, inappropriate stent choice (length, diameter, intrinsic characteristics such as rigidity), missed native vessel stenosis, inappropriate choice of location for placement, and excessive degree of extension of the stent into the IVC resulting in obstruction of contralateral flow. Therefore, appropriate stent selection and meticulous attention to precise placement with use of ancillary imaging (IVUS) should be employed to mitigate these risks.

Data regarding stent placement and long-term outcomes in pregnant patients are very limited. The most detailed study regarding management and short-term stent outcomes in pregnant patients is a small case series of 8 pregnancies in 6 women with previous stent placement, 3 of whom had MTS [56]. All patients received prophylactic dose anticoagulation, compression stockings, and were asked to sleep on their right side to avoid potential compression from the gravid uterus. Patients underwent follow-up ultrasound throughout the pregnancy. Stent compression or near occlusion was noted in the third trimester in 50% of patients. However, all cases of compression resolved without intervention postpartum. In an earlier series reported by the same authors, 3 patients had stent compression during pregnancy in the third trimester, and one developed re-thrombosis [57]. In another cohort of 310 women who underwent stenting, 12 women had subsequent pregnancies. Only one patient in this series developed subsequent stent stenosis, which occurred a year after her pregnancy, and was found during routine follow-up imaging. Management and surveillance during their subsequent pregnancies was not described [57]. Data on long-term stent outcomes following subsequent pregnancy are very limited. In one study of 29 patients, 22 of whom had MTS, primary patency rates ranged from 79–86% up to 120 months following placement, which is similar to what is reported in the general population [56].

Despite these observations, we do not recommend routine follow-up of stent patency during pregnancy in our patients, as stent compression alone would not be an indication for intervention. Imaging is instead prompted by new or worsening symptoms. We also do not advise altered sleeping habits, as it is unlikely that position can be significantly controlled during sleep.

### 3.4. Pharmacologic Management

#### Anticoagulation

Although anticoagulation is a cornerstone therapy for patients with thromboembolism, systemic anticoagulation as monotherapy is insufficient treatment for patients with MTS, given that it does not address the underlying compressive etiology [46,58,59]. PTS occurs in as many as 73% of patients with MTS treated with either anticoagulation alone [60,61] or in combination with thrombectomy [46]. Therefore, anticoagulation should be used as an adjuvant treatment to a more aggressive approach in these patients.

The anatomic changes throughout gestation, including decreased venous return secondary to caval compression of the gravid uterus, increased venous stasis, and an increased hepatic production of coagulation factors (VII, VIII, X and fibrinogen) all contribute to the increased incidence of VTE in pregnancy [62], which is exacerbated in those with MTS [63]. Clearly in the setting of acute thromboembolism anticoagulation is warranted, regardless of whether a stent is placed during the pregnancy. However, there is no consensus regarding the necessity for further anticoagulation following stent placement in the general population, and subsequently very little evidence to guide utilization of anticoagulation in pregnant patients who have a history of MTS with stent placement prior to pregnancy. Re-thrombosis was noted in one patient with prior stent placement prior to pregnancy, after missing only a single dose of prophylactic anticoagulation. Given the hypercoagulable state of pregnancy with high risk of recurrent thrombosis, and reports of stent compression during pregnancy, we favor adjuvant anticoagulation in all patients, with or without prior stent placement, and regardless of history of VTE. The risk of thrombosis is greatest in the weeks following delivery; therefore, more aggressive therapy with intermediate or adjusted dose anticoagulation could be considered for patients with additional risk factors.

Several organizations recommend consideration of timed delivery for patients on therapeutic anticoagulation, so as to minimize the duration of time without anticoagulation [30,58,64]. ACOG makes no specific recommendations in this regard, other than timing of withholding and restarting anticoagulation for the purpose of administration of anesthesia [11]. It is our practice to plan for a scheduled 39-week delivery for patients on anticoagulation, unless earlier delivery is warranted. Although not the primary focus of this study, anticoagulation in pregnant patients is accomplished with either LMWH or UFH [11,64]. LMWH can be continued until delivery and held with signs of labor or 12–24 h prior to scheduled delivery, depending upon the dose utilized. Although LMWH is generally favored, secondary to the ease of dosing, availability, and reduced risk of complications, such as bleeding and heparin induced thrombocytopenia, conversion to UFH late in gestation can be considered, based on preference of the managing team and patient [65,66]. UFH has a shorter half-life, can be held for a shorter period of time prior to delivery and administration of regional anesthesia, and can be more easily reversed in the case of bleeding risk. Both of these management strategies are accepted and recognized by ACOG [11].

For patients with acute thromboembolism during pregnancy (particularly late in pregnancy), history of thromboembolism despite anticoagulation, or in patients with history of multiple, recurrent thromboembolic events, we favor continuation of LMWH until admission, followed by initiation of an UFH infusion on admission, with continuation through latent labor, and re-initiation postpartum with transition to LMWH or bridge to warfarin in the postpartum period as the patient desires. This will preclude the ability to administer regional anesthesia; however, it minimizes the risk of recurrent thromboembolic event and/or clot propagation. Anticoagulation is then reinitiated following delivery based on guidelines from The Society for Obstetric Anesthesia and Perinatology [67]. It is our practice to individualize anticoagulation management after multidisciplinary discussion.

## 4. Conclusions

MTS is an underrecognized etiology contributing to thromboembolic disease in pregnant and postpartum patients. Secondary to physiologic compression of the LCIV by the gravid uterus and exaggerated lordosis, underlying MTS can enhance and hasten thromboembolic events, particularly with increasing gestational age. It is yet unknown as to what degree of compression results in clinically significant disease. Therefore, the diagnosis of MTS as the causative etiology for venous disease in pregnant patients can be challenging, if not impossible, and requires maintenance of high suspicion. Failure to recognize MTS can lead to significant undertreatment, resulting in long-term morbidity. Therefore, patients with suspected MTS as the underlying etiology for venous disease identified during pregnancy should have follow-up after delivery for definitive diagnosis and management. There are no specific criteria, nor are there universally accepted imaging tools for the diagnosis of MTS. Concern regarding radiation exposure is a major barrier to diagnostic and treatment options offered to pregnant patients. When feasible, ultrasound and non-contrast MR should be utilized when imaging is necessitated; however, radiation exposure associated with radiography, nuclear medicine, or CT is well below the threshold anticipated to cause fetal harm, with few exceptions. Therefore, when these modalities are necessary for logistic or diagnostic reasons, they should not be withheld from pregnant patients. Although Duplex ultrasound is considered the standard first line tool for identifying thromboses in pregnant patients, anatomic changes and difficulty imaging the iliofemoral vessels leads to diminished sensitivity. For these reasons, ancillary imaging tools, such as MRI/MRV or CT should be considered, particularly in patients with negative ultrasound despite high suspicion for disease.

Endovascular techniques and thrombolytic therapies have been safely performed in pregnant patients, at radiation dosage considered below the threshold for fetal harm and this type of risk/benefit ratio should be discussed with each patient prior to investigation. With appropriate counseling and employment of strategies to minimize radiation exposure these techniques may safely and successfully be utilized at any gestational age and should therefore not be withheld as a treatment option for this patient population when clinically indicated. In patients who do not have definitive therapy during pregnancy, the risk of recurrent thrombosis and PTS is high, and therefore these patients should be reevaluated following completion of pregnancy.

Three of our patients were diagnosed with MTS and had stents placed prior to pregnancy. Each was successfully managed with various anticoagulation strategies throughout pregnancy, without significant complications. Long-term data regarding stent outcomes in pregnant patients are limited. Although stent compression during pregnancy has been described, all cases had documented resolution following completion of pregnancy. Studies evaluating long-term patency in people who have become pregnant demonstrate similar rates to the general population. We therefore do not recommend routine follow-up of stent patency during pregnancy, as stent compression alone would not be an indication for intervention. Alternatively, imaging should be prompted by new or worsening symptom onset. Furthermore, these authors do not advise altered sleeping habits, as it is unlikely that position can be significantly controlled during sleep and there are no current data that would suggest an association with improved outcomes.

Patient 4 developed a pelvic vein thrombosis postpartum while on anticoagulation after missing a single dose of anticoagulant. It is well known that patients with MTS are at high risk for recurrent thromboembolism, particularly if the underlying compression is not addressed with stenting, as anticoagulation alone appears to be insufficient for management. Given the thrombogenic nature of both pregnancy and MTS, we recommend anticoagulation and aspirin for these patients during pregnancy and the postpartum period, even following iliocaval stent placement, secondary to description of stent compression during gestation. We also encourage patients to wear compression stockings to minimize symptoms associated with venous insufficiency. We favor timed delivery to minimize the time without anticoagulation. More aggressive management with continued anticoagulation during the latent phase of labor, and higher anticoagulation doses postpartum, should be considered for patients with acute thromboembolism during pregnancy or those with history of recurrent thromboembolic events or additional risk factors.

We monitor patients closely throughout pregnancy and the postpartum period in our multidisciplinary heart disease in pregnancy clinic where patients are followed by both maternal fetal experts as well as cardiology and venous disease experts. After pregnancy, these women should have long-term follow-up with a vein specialist. Even in patients with a history of definitive treatment, young age and reproductive history can play a large role in subsequent development of complications including re-occlusion and PTS. With appropriate anticoagulation, and ideally, with close observation in a multidisciplinary team setting, patients with MTS can be safely and successfully managed during pregnancy and the postpartum period.

## Figures and Tables

**Table 1:** Summary of cases. All patients were managed with anticoagulation, aspirin, and compression stockings. All patients delivered at term, without obstetric complications; however, one patient developed a postpartum gonadal vein thrombosis after missing a dose of her anticoagulant. Abbreviations: GP: gravidity parity, GA: gestational age, DVT: deep venous thrombosis, LCFV: left common femoral vein, LCIV: left common iliac vein, IVC: inferior vena cava, MTS: May Thurner Syndrome, UFH: unfractionated heparin, CDT: catheter-directed thrombolysis, LSFV: left superficial femoral vein, LMWH: low molecular weight heparin, PP: postpartum, ASA: aspirin, SVD: spontaneous vaginal delivery, US: ultrasound, IOL: induction of labor, IVUS: intravenous ultrasound, LEIV: left external iliac vein. * a: index pregnancy, ^‡^ b: subsequent pregnancy.

Case/Age	GP	History	GA at MTS Diagnosis	Diagnostic Modality	Management	Obstetric Outcome	Complications (Obstetric/Venous)
**1/29**	G2P2		9	Doppler/Duplex US (DVT LCFV to tibial), MRI/A/V (DVT extending from IVC, LCIV, LCFV, c/w MTS), venography (MTS)	UFH, CDT, balloon angioplasty of LCIV and LSFV. Adjusted dose LMWH, continued through 8 wks PP, ASA, compression stockings. LCIV stent PP.	SVD at term	None
2a */29	G1P1	h/o DVT, known MTS with h/o LCIV stent	NA	Initial diagnosis with Duplex US (MTS)	Adjusted dose LMWH, transitioned to UFH, ASA, compression stockings. LMWH continued for 6 wks PP with ASA.	IOL at 39 wks, SVD Female 3690 g (83%)	None
**2b ^‡^/32**	G2P2		NA		Adjusted dose LMWH, transitioned to UFH, ASA, compression stockings. LMWH continued for 6 wks PP with ASA.	IOL at 40 wks, SVD Male 4470 g (98%)	None
**3/33**	G2P2	Known MTS with h/o LCIV stent, followed by complete stent occlusion	NA	Initial diagnosis with Duplex US, venogram/IVUS (MTS)	Adjusted dose LMWH, transitioned to UFH, ASA, compression stockings. LMWH bridged to warfarin postpartum.	IOL at 39, SVD Male 3060 g (27%)	None
**4/33**	G4P4	Pelvic/perineal varicosities	3	Duplex US (pelvic congestion, reflux and collaterals), venogram/IVUS (MTS)	LCIV and LEIV stents placed early pregnancy (prior to knowledge of pregnancy). Prophylactic LMWH transitioned to UFH, ASA, compression stockings. Prophylactic LMWH PP.	SVD at 39 Male, 3593 g (69%)	Gonadal v. thrombosis 6 d PP, (single missed dose of LMWH)

## Data Availability

Not applicable.
